# Under the spotlight: social anxiety and music performance anxiety in solo-exposed wind instrumentalists compared to section players in orchestra settings

**DOI:** 10.3389/fpsyg.2026.1777719

**Published:** 2026-06-24

**Authors:** Shuai Yang, Heng Chen, Guang Yang

**Affiliations:** 1 Zhengzhou University, Zhengzhou, China; 2 University of Malaya, Kuala Lumpur, Malaysia; 3 Zhejiang Conservatory of Music, Hangzhou, China

**Keywords:** exposure level, mediation, moderation, music performance anxiety, orchestra, social anxiety, wind instrumentalists

## Abstract

Music performance anxiety (MPA) is a prevalent psychological phenomenon affecting musicians at all levels. While prior research has established that solo performance contexts typically elicit higher anxiety than ensemble settings, less attention has been paid to variations in exposure levels within orchestra environments. This cross-sectional study examined the association between exposure level and MPA among wind instrumentalists in orchestra settings, investigating the mediating role of social anxiety and the moderating role of performance experience. A total of 152 wind instrumentalists were classified into either the solo-exposed group (section principals or those performing solo passages; *n* = 74) or the section group (regular section players; *n* = 78). Participants completed the Kenny Music Performance Anxiety Inventory (K-MPAI), Liebowitz Social Anxiety Scale (LSAS), and State–Trait Anxiety Inventory-Trait subscale (STAI-T). Results indicated that solo-exposed players reported significantly higher MPA (*d* = 0.44) and social anxiety (*d* = 0.38) compared to section players. Mediation analysis revealed that social anxiety significantly mediated the statistical relationship between exposure type and MPA, accounting for 44.98% of the total effect. Given the cross-sectional design, these findings indicate associations and the direction of effects should be interpreted with caution. Furthermore, performance experience moderated the relationship between exposure type and MPA, with the between-group difference being significant only among less experienced players. Subscale analyses demonstrated that group differences were primarily evident in performance-situation anxiety and worry/rumination dimensions rather than early relationship factors or psychological vulnerability. These findings extend the understanding of context-specificity in MPA and provide empirical evidence for targeted interventions for high-exposure musicians in orchestra settings.

## 1 Introduction

Music performance anxiety (MPA) is a prevalent and consequential psychological phenomenon affecting musicians at all skill levels (Fernholz et al., 2019; Kenny, 2011). Severe MPA not only impairs performance quality but may also lead to career disruption, diminished quality of life, and secondary problems such as substance abuse (Kenny, 2011). Recent evidence further indicates that MPA exerts multidimensional negative effects on music students’ academic performance, physical health, and psychological well-being (Hernández-Dionis et al., 2025). Therefore, gaining a deeper understanding of the factors influencing MPA and their underlying mechanisms is of considerable significance for maintaining musicians’ psychological well-being and supporting their professional development.

Previous research has established that solo performance contexts typically elicit higher anxiety levels than ensemble settings (Papageorgi et al., 2013). However, this dichotomy may be overly simplistic. According to cognitive models of social anxiety, anxiety intensity is largely determined by the degree to which individuals perceive themselves as the focus of evaluative attention (Rapee and Heimberg, 1997). Within the same orchestra, performers occupy positions with substantially different levels of visibility: section principals and those performing solo passages become identifiable focal points of audience scrutiny, whereas regular section members remain relatively anonymous within the collective sound. This within-ensemble variation in “degree of exposure”—defined here as the extent to which a performer’s individual contribution is singled out for evaluative attention—and its effects on MPA have yet to be systematically investigated.

Wind instrumentalists represent an ideal population for examining this issue, given their smaller section sizes, frequent assignment of exposed solo passages, and distinctive vulnerability to anxiety-related physiological interference with breath control (see Literature Review for detailed discussion). Despite these considerations, specialized research on MPA among wind instrumentalists remains relatively limited.

The present study aimed to examine the association between exposure level and MPA among wind instrumentalists in orchestra settings and to explore its psychological mechanisms. Specifically, wind instrumentalists were classified as either “solo-exposed” (section principals or those performing solo passages) or “section players” (regular section members), and group differences in MPA and social anxiety were examined. Additionally, this study explored the mediating role of social anxiety and the moderating effect of performance experience. The findings are expected to advance theoretical understanding of the context-specificity of MPA and provide empirical evidence for orchestra management and psychological health interventions for performers.

## 2 Literature review

### 2.1 Conceptualization and measurement of music performance anxiety

Music performance anxiety (MPA) refers to persistent, marked anxious apprehension experienced in public performance situations, the severity of which is sufficient to impair performance quality and professional development (Kenny, 2011). Core features of MPA include cognitive components (e.g., catastrophic thinking, fear of failure), physiological components (e.g., elevated heart rate, trembling hands, dry mouth), and behavioral components (e.g., performance avoidance, diminished performance quality). Epidemiological research indicates that MPA affects approximately 15–25% of professional musicians, with even higher rates of 30–40% among music students (Fernholz et al., 2019).

Kenny (2011) proposed a three-type taxonomy of MPA, distinguishing among three subtypes: (1) focal MPA, primarily associated with fear of social evaluation in performance situations and highly overlapping with social anxiety disorder; (2) panic-type MPA, characterized by sudden panic attacks that may occur before or during performance; and (3) generalized MPA, associated with generalized anxiety and early adverse experiences, manifesting as a more diffuse anxiety tendency. This classification framework is valuable for understanding the heterogeneity of MPA and provides a theoretical basis for identifying differential intervention needs across subtypes. Independently, Spahn et al. (2021) employed cluster analysis to identify empirically derived subgroups of musicians based on anxiety profiles. Although their data-driven clusters do not directly correspond to Kenny’s theoretically derived taxonomy, both approaches converge on the broader conclusion that MPA is not a unitary construct but encompasses qualitatively distinct subtypes, underscoring the importance of differentiated assessment and intervention.

Regarding measurement instruments, the Kenny Music Performance Anxiety Inventory (K-MPAI) is currently the most widely used MPA-specific scale. The K-MPAI was developed based on Barlow (2000) triple vulnerability model of emotional disorders and comprises 40 items across six dimensions: early relationship context, psychological vulnerability, proximal somatic anxiety, depression/hopelessness, performance-situation anxiety, and worry/rumination. The scale has been translated and validated across multiple languages and cultural contexts, demonstrating sound psychometric properties (Kenny, 2023).

### 2.2 Relationship between music performance anxiety and social anxiety

MPA and social anxiety share close conceptual and empirical associations. From a diagnostic perspective, the DSM-5 classifies MPA under the “performance-only” specifier of social anxiety disorder (American Psychiatric Association, 2013), acknowledging the overlap in core features between the two constructs—both center on fear of negative evaluation and are accompanied by avoidance behavior and significant functional impairment.

Empirical research supports this association. Cox and Kenardy (1993) found that social phobia scale scores showed moderate to strong correlations with MPA severity in a sample of instrumental music students. Gorges et al. (2007) demonstrated that social anxiety correlated highly with MPA in a sample of 142 musicians and that the performance anxiety subscale of social anxiety specifically predicted MPA severity, independent of general anxiety traits. More recently, Dobos et al. (2019) confirmed significant positive correlations between social phobia and MPA in a sample of Hungarian musicians, with females reporting higher levels of both MPA and social anxiety. Separately, Osborne and Kenny (2008) found that trait anxiety was one of the strongest predictors of MPA in adolescent musicians, further indicating the role of anxiety-related dispositions in MPA vulnerability. More recent investigations have confirmed that MPA shares significant overlap with other anxiety disorders while maintaining distinct features (Wiedemann et al., 2022).

Nevertheless, MPA and social anxiety are not entirely equivalent. Some researchers have noted that MPA is domain-specific—certain performers may experience severe anxiety in musical performance situations while functioning normally in everyday social contexts (Kenny, 2011). Furthermore, MPA involves factors that are not fully captured by social anxiety frameworks, such as concerns about technical errors, uncertainty about musical interpretation, and physical symptoms specific to particular instruments. Therefore, it may be more appropriate to view social anxiety as an important risk factor or mediating mechanism for MPA rather than an equivalent concept. Indeed, recent studies have highlighted the interplay between MPA, perfectionism, and fear of negative evaluation in conservatory musicians (Gök and Yalçınkaya-Alkar, 2024).

### 2.3 Effects of performance context on music performance anxiety

Performance context is an important external factor influencing MPA. Research indicates that solo performance contexts typically elicit higher anxiety levels than ensemble settings (Papageorgi et al., 2013). This difference can be understood from multiple perspectives: soloists bear full artistic responsibility, cannot “hide” within a group, and their mistakes are more readily identified by audiences; in contrast, ensemble settings provide a degree of “social cover,” allowing performers to distribute attention across the collective sound rather than focusing solely on individual performance.

Robson and Kenny (2017) refined this understanding, finding that even within the ensemble framework of symphony orchestras, performers in different positions experience varying anxiety levels. Players in principal positions (e.g., section principals) reported higher MPA, suggesting that “degree of exposure” may be a more nuanced analytical dimension than the solo-versus-ensemble dichotomy. However, that study did not systematically operationalize the concept of exposure level or explore its underlying psychological mechanisms.

Other characteristics of performance contexts have also been found to correlate with MPA, including audience size and composition (peers vs. general audience), venue formality, presence of evaluation or competition, and difficulty and familiarity of repertoire (Papageorgi et al., 2007). Furthermore, research has demonstrated that performance context influences not only anxiety levels but also flow experiences, with audience presence and MPA interacting to affect performers’ psychological states (Guyon et al., 2022; Cohen and Bodner, 2021). These findings collectively indicate that MPA reflects not only individual traits but also the interaction between the individual and the situation. To integrate these observations theoretically, the present study draws on the self-presentation model of social anxiety (Schlenker and Leary, 1982) and the cognitive model of social phobia (Rapee and Heimberg, 1997). According to these frameworks, anxiety arises when individuals are motivated to make a favorable impression on an audience but doubt their ability to do so, and when they perceive themselves as the object of evaluative scrutiny. Within orchestras, the degree of exposure determines the extent to which these conditions are met: solo-exposed performers—section principals and those performing solo passages—are individually identifiable, bear primary artistic responsibility for exposed moments, and perceive heightened evaluative scrutiny from audiences and colleagues. In contrast, section players benefit from relative anonymity within the collective texture, reducing both the salience of individual evaluation and the perceived consequences of error. This conceptualization positions exposure level as a situational variable that modulates the core cognitive antecedents of social anxiety and, by extension, MPA. Within Barlow (2000) triple vulnerability model, exposure level functions as a situation-specific trigger that interacts with generalized biological and psychological vulnerabilities to produce anxiety responses, corresponding to the third layer of vulnerability in Barlow’s framework.

### 2.4 Unique characteristics of wind instrumentalists

Wind instrumentalists occupy a distinctive position within orchestras, potentially exposing them to particular anxiety risks. Unlike string sections (which typically have multiple players per part), wind sections have smaller personnel—often only 2–4 players per section—meaning that each wind player’s individual contribution is more prominent in the overall sound (Sternbach, 2008). Research has confirmed that pre-performance anxiety, catastrophizing, and bodily complaints vary significantly by instrument type (Sokoli et al., 2022).

More importantly, wind sections frequently carry signature solo passages in symphonic works. The oboe’s tuning responsibility, cadenzas for flute and clarinet, heroic themes for horn, and fanfare-like proclamations for trumpet—these are all “exposed moments” in the symphonic literature during which performers are highlighted from the ensemble and become the audience’s focal point. For those serving as section principals or regularly performing such passages, the psychological experience may more closely resemble that of soloists despite being in an orchestra setting (Parasuraman and Purohit, 2000).

The physiological characteristics of wind performance may also elevate anxiety risk. Unlike string performance, wind playing is highly dependent on breath control and fine coordination of embouchure muscles; anxiety-induced physiological responses (e.g., rapid breathing, dry mouth, lip trembling) directly interfere with these functions, creating a vicious cycle of “anxiety—physiological interference—impaired performance—heightened anxiety” (Yoshie et al., 2009). This direct coupling of somatic symptoms and performance technique makes wind players particularly sensitive to the somatic components of anxiety. Studies have documented specific respiratory patterns including increased variability and sighing in high-anxious music students during performance contexts (Guyon et al., 2020a). Similar somatic–psychological interactions have been observed among singers, who share the dependence on breath control with wind players. Notably, Cui et al. (2022) found that subjective MPA did not significantly correlate with physiological stress indicators during opera performances, whereas trait anxiety and experience were significant predictors, suggesting that the relationship between subjective anxiety and objective physiological arousal may be more complex than commonly assumed. Physiological research has also begun to characterize cardiovascular responses during wind and vocal performance, documenting heart rate variability patterns during flute playing and singing (Harmat and Theorell, 2010) and cardiovascular reactivity under audition stress conditions (Chanwimalueang et al., 2017), providing a foundation for understanding the physiological dimension of performance anxiety in these populations.

Despite these unique characteristics, specialized research on MPA among wind instrumentalists remains relatively limited. Existing literature has predominantly focused on the broad category of “orchestral musicians” or has analyzed wind instruments together with other instrument types, without adequately addressing heterogeneity within the wind player population. Notable exceptions include pilot studies examining intervention approaches specifically for wind musicians (Blasco-Lafarga et al., 2022).

### 2.5 Performance experience and music performance anxiety

The influence of performance experience on MPA is a topic that has received considerable attention but with inconsistent conclusions. From a theoretical standpoint, repeated successful performance experiences should reduce threat appraisal of performance situations through habituation mechanisms while enhancing self-efficacy through mastery experiences, thereby alleviating anxiety symptoms (Bandura, 1977).

Some empirical studies support this expectation. Studer et al. (2011) found a negative correlation between performance experience and MPA in a large sample of music students, with highly experienced individuals reporting lower anxiety levels. From a challenge-threat perspective, experienced performers may appraise high-stakes situations as challenges rather than threats, leading to more adaptive psychophysiological responses (Guyon et al., 2020b). Experimental evidence further supports the habituation hypothesis: Candia et al. (2023) demonstrated that repeated stage exposure significantly reduced heart rate, self-reported restlessness, and playing errors across three consecutive public performances, providing direct evidence that accumulating performance experience can attenuate the physiological and behavioral manifestations of MPA.

However, other studies present a more complex picture. Kenny et al. (2014) found no significant correlation between years of playing and MPA among professional orchestra musicians, suggesting that accumulated experience does not necessarily lead to reduced anxiety. Steptoe (2001) even reported increased MPA among some senior performers, possibly related to career burnout, pressure to maintain reputation, or accumulated negative performance experiences. Recent mediation studies have identified emotional intelligence and self-efficacy as key mechanisms through which social support buffers MPA among music students (Zhang and Jenatabadi, 2024). Similarly, Yang et al. (2025) found that psychological resilience moderated the relationship between professional performance competence and MPA, indicating that individual psychological resources may buffer the anxiety-inducing effects of situational demands.

These contradictory findings suggest that the relationship between performance experience and MPA may be nonlinear or moderated by other variables. For example, the “quality” of experience (successful vs. unsuccessful) may be more important than “quantity”; the effects of experience may also vary by type of performance context—experience accumulated in low-pressure settings may not transfer to high-pressure situations. Furthermore, performers with different exposure levels may benefit from experience to varying degrees, a potential moderating effect that has yet to be systematically examined.

### 2.6 Research questions and hypotheses

Synthesizing the above literature, the following gaps exist in current research: first, most studies have adopted a solo-versus-ensemble dichotomy, with less attention to variations in exposure level within ensemble settings; second, the role of social anxiety as a mediating mechanism linking exposure level to MPA has not been tested; third, the moderating role of performance experience—particularly whether experience can buffer the anxiety risks associated with high-exposure positions—remains to be explored; and fourth, wind instrumentalists as a population with unique exposure characteristics have not been specifically studied.

**Figure 1:**
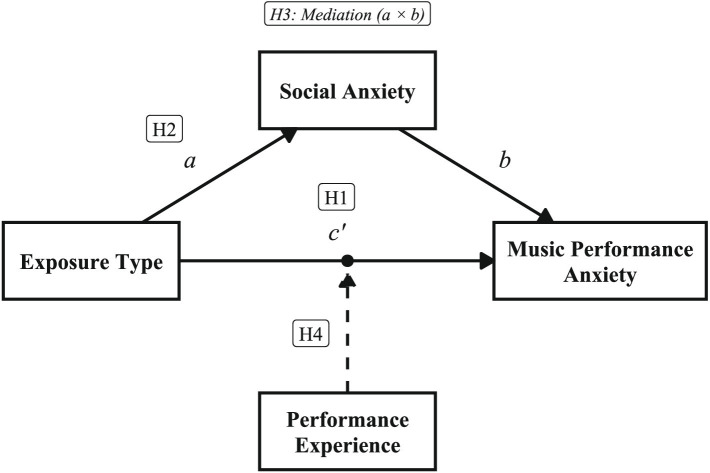
Hypothesized moderated mediation model. H1 = direct effect of exposure type on MPA; H2 = effect of exposure type on social anxiety; H3 = mediation effect through social anxiety; H4 = moderation effect of performance experience.

To address these gaps, the present study focused on wind instrumentalists in orchestra settings, classifying them as either “solo-exposed” (section principals, those performing solo passages) or “section players” (regular section members), and systematically examined the association between exposure level and MPA, the mediating role of social anxiety, and the moderating role of performance experience. The theoretical model is presented in Figure 1, and the following hypotheses were proposed:

*H1*: Solo-exposed wind instrumentalists will exhibit significantly higher MPA levels than section players.

*H2*: Solo-exposed wind instrumentalists will exhibit significantly higher social anxiety levels than section players.

*H3*: Social anxiety will mediate the relationship between exposure type and MPA.

*H4*: Performance experience will moderate the relationship between exposure type and MPA, such that the between-group difference in MPA will be smaller among more experienced players.

## 3 Method

### 3.1 Study design

This study employed a cross-sectional comparative design to examine differences in MPA and social anxiety between “solo-exposed” and “section” wind instrumentalists in orchestra settings, and to explore the mediating role of social anxiety and the moderating effect of performance experience. A quantitative approach was adopted, with data collected through standardized questionnaires.

### 3.2 Participants

#### 3.2.1 Sample recruitment

Participants were recruited through multiple channels: (1) partnerships with professional symphony orchestras and philharmonic orchestras in China, distributing recruitment information to wind section players; (2) recruitment through ensembles affiliated with professional music conservatories and high-level amateur orchestras; (3) recruitment announcements through professional organizations such as the Wind Music Society of the Chinese Musicians’ Association; and (4) expanded recruitment via WeChat official accounts and Douban groups targeting musician communities.

#### 3.2.2 Inclusion and exclusion criteria

Inclusion criteria: (1) aged 18 to 65 years; (2) majoring in woodwind instruments (flute, oboe, clarinet, bassoon) or brass instruments (horn, trumpet, trombone, tuba); (3) at least 1 year of orchestra playing experience; (4) public performance experience within the past 6 months.

Exclusion criteria: (1) currently receiving psychiatric treatment or psychological counseling; (2) initiated anxiolytic, antidepressant, or other psychotropic medication within the past 6 months; (3) severe hearing impairment affecting normal orchestra performance.

#### 3.2.3 Group classification criteria

Participants were classified into the solo-exposed group or section group based on their degree of exposure within the orchestra.

Solo-exposed group: Meeting any of the following criteria: (1) currently serving as section principal (principal/first chair) in their orchestra; (2) performed solo passages in orchestral works three or more times in the past 12 months; (3) assigned technically demanding passages requiring independent display, such as the oboe’s orchestral tuning or signature solo passages for horn.

Section group: Meeting all of the following criteria: (1) holding a non-principal position within the section; (2) no solo passage performances or only one or fewer in the past 12 months; (3) participating primarily in ensemble playing without requirements for independent display.

For borderline cases, two researchers independently made classifications, with disagreements resolved through discussion. If a participant experienced a position change within the past 12 months, classification was based on their current position.

#### 3.2.4 Sample size estimation

Based on effect sizes from prior research comparing MPA in solo versus ensemble contexts (Cohen’s *d* ≈ 0.5), with *α* = 0.05 and statistical power (1-*β*) = 0.80, G*Power 3.1 was used to estimate a required sample of 64 participants per group. Accounting for approximately 10% data attrition, the target total sample size was set at no fewer than 140 participants.

### 3.3 Measures

#### 3.3.1 Kenny music performance anxiety inventory (K-MPAI)

The 40-item revised Kenny Music Performance Anxiety Inventory was used to measure participants’ MPA levels. Items are rated on a 7-point Likert scale (0 = “strongly disagree” to 6 = “strongly agree”), with total scores ranging from 0 to 240; higher scores indicate higher anxiety levels. The K-MPAI comprises six subscales: early relationship context, psychological vulnerability, proximal somatic anxiety, depression/hopelessness, performance-situation anxiety, and worry/rumination. The scale has been validated across musician populations in multiple countries (Kenny, 2023).

At the time the present study was designed and data were collected, no peer-reviewed psychometric validation of the K-MPAI in a mainland Chinese instrumentalist sample had been published. Kenny (2023) cross-cultural review identified the Simplified Chinese rendering only as an unpublished personal-communication translation, and prior Chinese-context empirical studies (e.g., Cui et al., 2024) had used informal Chinese renderings without reporting formal psychometric validation. Accordingly, with written permission from the original author (D. T. Kenny, Personal Communication, January 2025), the research team independently translated the K-MPAI into Mandarin Chinese for the present study, following standard translation-back-translation procedures (Brislin, 1970). Specifically, the first and second authors—both bilingual researchers with backgrounds in music and music psychology—independently translated the K-MPAI from English into Mandarin Chinese. The third author, also bilingual and blind to the original English version, then back-translated the Chinese version into English. Discrepancies between the back-translation and the original were resolved through panel discussion involving all translators. A subsequent pilot test with 15 wind instrumentalists assessed item clarity and cultural appropriateness, after which minor wording adjustments were made. Subsequent to our data collection, Pan et al. (2025) published the first peer-reviewed psychometric validation of a Chinese K-MPAI in a sample of vocal-music students, yielding a reduced four-factor 26-item solution; however, the authors explicitly cautioned that their validation does not generalize to instrumentalists, given established differences between vocal and instrumental performers. To our knowledge, no published psychometric validation of a Chinese K-MPAI in a wind-instrumentalist sample currently exists. In the present sample, the full 40-item Chinese K-MPAI demonstrated high internal consistency (Cronbach’s *α* = 0.92).

#### 3.3.2 Liebowitz social anxiety scale (LSAS)

The Liebowitz Social Anxiety Scale (LSAS; Liebowitz, 1987) was used to measure participants’ social anxiety levels. The scale comprises 24 items covering social interaction situations and performance/observation situations. Each item is rated on two dimensions: fear (0–3) and avoidance (0–3), yielding fear subscale, avoidance subscale, and total scores (0–144). The present study used the standardized Chinese version translated and validated by He and Zhang, 1999, which has demonstrated satisfactory reliability and validity in Chinese populations.

#### 3.3.3 State–trait anxiety inventory-trait subscale (STAI-T)

The Trait Anxiety subscale of the State–Trait Anxiety Inventory (STAI-T; Spielberger et al., 1983) was used to measure participants’ relatively stable anxiety tendencies. This subscale comprises 20 items rated on a 4-point Likert scale (1 = “almost never” to 4 = “almost always”), with total scores ranging from 20 to 80. The present study used the Chinese version validated by Wang et al., 1999, which has been widely applied in Chinese psychological research with established psychometric properties.

#### 3.3.4 Demographic and musical background questionnaire

A researcher-developed questionnaire collected the following information: (1) demographic information: age, gender, highest educational attainment, marital status; (2) musical training background: primary instrument, years of study, whether formal professional training was received; (3) orchestra experience: orchestra type, years of playing, current position; (4) performance experience: number of performances in the past 12 months, number of solo performances; (5) anxiety management strategies: beta-blocker use and other coping methods.

### 3.4 Procedure

#### 3.4.1 Ethical approval

All participants read the informed consent form before completing the questionnaire, understood the study purpose, procedures, and confidentiality principles, and confirmed informed consent via electronic signature. Research data were coded to ensure anonymity and confidentiality.

#### 3.4.2 Data collection

Data collection proceeded in three phases:

Phase 1 (Recruitment and Screening): Recruitment information was disseminated through the above channels. Interested individuals completed a pre-screening questionnaire, and the research team identified eligible participants and preliminarily assigned group classifications.

Phase 2 (Formal Assessment): Formal questionnaire links were sent to eligible participants and administered via the Wenjuanxing platform. The questionnaire sequence was: informed consent form, demographic and musical background questionnaire, K-MPAI, LSAS, and STAI-T. The presentation order of the three standardized scales was randomized to control for order effects; estimated completion time was approximately 20–25 min.

Phase 3 (Data Cleaning): Questionnaires with completion rates below 80% were excluded, and invalid responses (e.g., straight-line responding) were identified and removed. Group assignments were verified, and the final valid sample was determined.

Data were collected between February and April 2025, scheduled outside the primary performance season to minimize the immediate influence of performance season workload on anxiety levels.

### 3.5 Data analysis

#### 3.5.1 Preliminary analyses

Descriptive statistics were computed for all variables, including means, standard deviations, ranges, skewness, and kurtosis. Normality was assessed using Shapiro–Wilk tests, homogeneity of variance using Levene’s tests, and internal consistency using Cronbach’s *α* coefficients for each scale. Missing data were handled as follows: mean substitution for <5% missing, multiple imputation for 5–20% missing, and case deletion considered for >20% missing. Pearson correlation coefficients were calculated among primary variables.

#### 3.5.2 Primary analyses

H1 and H2 (Between-Group Differences): Independent samples *t*-tests were used to compare between-group differences on K-MPAI and LSAS total and subscale scores; Mann–Whitney U tests were used when normality assumptions were not met. ANCOVA was subsequently conducted with age, gender, instrument type, years of playing, and STAI-T as covariates to examine whether group differences remained significant after controlling for these variables. Cohen’s *d* and 95% confidence intervals were calculated.

H3 (Mediation Effect): PROCESS macro Model 4 was used to test the mediating role of social anxiety in the relationship between exposure type and MPA. Exposure type served as the independent variable (section = 0, solo-exposed = 1), LSAS total score as the mediator, and K-MPAI total score as the dependent variable. Bootstrap methods (5,000 resamples) were used to estimate indirect effects and 95% confidence intervals; mediation was considered significant if the confidence interval did not include zero. Path coefficients (a, b, c, c’) and the proportion mediated were reported.

H4 (Moderation Effect): Hierarchical regression analysis was used to test the moderating role of performance experience (years of orchestra playing). Step 1 entered control variables; Step 2 entered mean-centered exposure type and performance experience; Step 3 entered the interaction term. A significant interaction term indicated moderation, followed by simple slopes analysis to examine the effect of exposure type on MPA at high experience (M + 1SD) and low experience (M - 1SD) levels.

#### 3.5.3 Supplementary analyses

Subgroup analyses by instrument type were conducted, examining between-group differences separately among woodwind and brass players, and using two-way ANOVA to examine the interaction effect between exposure type and instrument type. Between-group comparisons were conducted for the six K-MPAI subscales with Bonferroni correction (*α* = 0.008).

#### 3.5.4 Statistical software

Statistical analyses were conducted using SPSS 26.0, with mediation and moderation analyses conducted using PROCESS macro v4.0. The significance level was set at α = 0.05 unless otherwise specified.

## 4 Results

### 4.1 Participant characteristics

**Figure 2:**
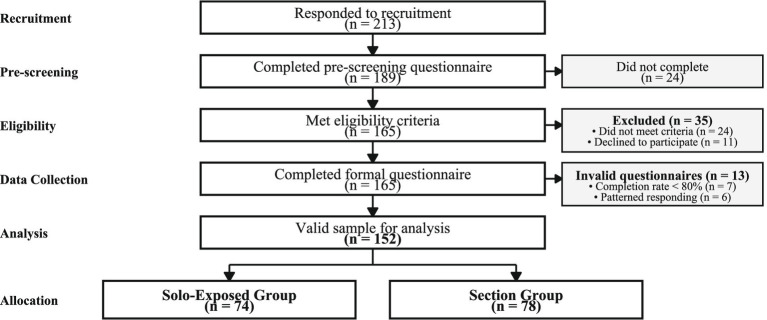
Participant recruitment and screening flowchart.

The participant recruitment and screening flowchart is presented in Figure 2. Initially, 213 individuals responded to recruitment, with 189 completing the pre-screening questionnaire. Of these, 24 were excluded for not meeting inclusion criteria or meeting exclusion criteria. Among the 165 eligible individuals, 11 initially agreed but later declined to participate before completing the formal questionnaire; however, replacement participants were recruited to maintain the target sample size. Ultimately, 165 individuals completed the formal questionnaire; after excluding 13 invalid questionnaires (7 with completion rates below 80%, 6 with obvious patterned responding), a valid sample of 152 participants was obtained, comprising 74 in the solo-exposed group and 78 in the section group.

Demographic and musical background characteristics for both groups are presented in Table 1. The overall sample had a mean age of 28.34 years (*SD* = 7.82), with 46.1% female. Regarding instrument type distribution, 53.9% were woodwind players and 46.1% were brass players. Participants had a mean of 8.67 years (*SD* = 5.43) of orchestra playing experience and a mean of 18.25 performances (*SD* = 12.38) in the past 12 months.

**Table 1:** Comparison of demographic and musical background characteristics between groups.

**Variable**	**Solo-exposed (*n* = 74)**	**Section (*n* = 78)**	***t*/χ** ^**2**^	***p***
Demographics				
Age *M* (SD)	28.92 (8.15)	27.79 (7.48)	0.87	0.386
Female *n* (%)	32 (43.2%)	38 (48.7%)	0.42	0.518
Bachelor’s or higher *n* (%)	68 (91.9%)	70 (89.7%)	0.21	0.647
Musical background				
Instrument type (brass) n (%)	31 (41.9%)	39 (50.0%)	1.15	0.284
Years playing M (SD)	9.15 (5.68)	8.21 (5.17)	1.23	0.221
Performances past 12 mo M (SD)	19.82 (13.24)	16.76 (11.35)	1.58	0.116
Solo passages past 12 mo M (SD)	5.82 (3.15)	0.58 (0.72)	13.87	<0.001
Beta-blocker use n (%)	21 (28.4%)	11 (14.1%)	4.58	0.032

M = mean; SD = standard deviation; independent samples *t*-tests for continuous variables, chi-square tests for categorical variables.

Between-group comparisons indicated no significant differences in age (*t* = 0.87, *p* = 0.386), gender distribution (χ^2^ = 0.42, *p* = 0.518), instrument type (χ^2^ = 1.15, *p =* 0.284), or years of orchestra playing (*t* = 1.23, *p* = 0.221), indicating comparable baseline characteristics between groups. As expected, the solo-exposed group had significantly more solo passage performances in the past 12 months (*M* = 5.82, *SD* = 3.15) compared to the section group (*M* = 0.58, *SD* = 0.72), *t*(150) = 13.87, *p* < 0.001, consistent with group classification criteria. Exploratory analysis revealed that beta-blocker use was higher in the solo-exposed group (28.4%) than in the section group (14.1%), χ^2^ = 4.58, *p* = 0.032, consistent with the expectation that high-exposure performers may have greater pharmacological management needs.

### 4.2 Descriptive statistics

Descriptive statistics and the correlation matrix for primary variables are presented in Table 2. The overall sample K-MPAI mean was 90.12 (*SD* = 37.54), LSAS mean was 48.56 (*SD* = 21.83), and STAI-T mean was 42.47 (*SD* = 9.76). All scales demonstrated good internal consistency, with Cronbach’s *α* coefficients of 0.92 for K-MPAI, 0.89 for LSAS, and 0.87 for STAI-T.

**Table 2:** Descriptive statistics and correlation matrix.

Variable	*M*	SD	P25	P50	P75	*α*	1	2	3	4
1. K-MPAI	90.12	37.54	65	88	115	0.92	—			
2. LSAS	48.56	21.83	34	47	63	0.89	0.58***	—		
3. STAI-T	42.47	9.76	36	42	49	0.87	0.65***	0.52***	—	
4. Years playing	8.67	5.43	4	7	12	—	−0.18*	−0.12	−0.09	—

N = 152; M = mean; SD = standard deviation; α = Cronbach’s α. *p < 0.05, **p < 0.01, ***p < 0.001.

Correlation analyses revealed significant positive correlations between K-MPAI and LSAS total scores (*r* = 0.58, *p <* 0.001) and between K-MPAI and STAI-T (*r* = 0.65, *p <* 0.001), indicating moderate to strong associations among MPA, social anxiety, and trait anxiety. LSAS and STAI-T were also significantly positively correlated (*r* = 0.52, *p <* 0.001). Years of orchestra playing showed a weak negative correlation with K-MPAI (*r* = −0.18, *p =* 0.026), suggesting that performance experience may be associated with lower MPA levels, though this association was modest.

To contextualize these scores against established benchmarks, LSAS total scores were interpreted using published clinical severity criteria (Liebowitz, 1987): scores of 0–29 indicate non-significant social anxiety, 30–49 moderate, 50–64 marked, 65–79 severe, and ≥80 very severe. The overall sample mean (*M* = 48.56) fell in the moderate range. Notably, the solo-exposed group mean (*M* = 52.78) approached the threshold for marked social anxiety, while the section group mean (*M* = 44.56) remained in the moderate range. Regarding MPA, using the suggested clinical threshold of 105 on the K-MPAI (Kenny, 2011), 41.9% of solo-exposed players (*n* = 31) and 25.6% of section players (*n* = 20) scored at or above this level, a difference that was statistically significant, χ^2^(1) = 4.52, *p =* 0.033, suggesting that a substantially greater proportion of high-exposure players experienced clinically significant MPA. STAI-T scores for both groups fell within the range typically reported for non-clinical adult populations in Chinese normative studies (*M* ≈ 38–42), indicating that the sample did not exhibit elevated general trait anxiety relative to the general population.

### 4.3 Primary results

#### 4.3.1 Between-group differences in music performance anxiety

H1 predicted that the solo-exposed group would have significantly higher MPA levels than the section group. Independent samples *t*-test results indicated that the solo-exposed group had significantly higher K-MPAI total scores (*M* = 98.47, *SD* = 38.26) compared to the section group (*M* = 82.20, *SD* = 35.18), *t*(150) = 2.82, *p =* 0.005, Cohen’s *d* = 0.44, 95% CI [0.12, 0.77]. This effect size represents a medium effect.

ANCOVA was subsequently conducted with age, gender, instrument type, years of playing, and STAI-T as covariates. Results showed that the between-group difference remained significant after controlling for these variables, *F*(1, 144) = 5.23, *p =* 0.024, η^2^*p =* 0.035. Notably, the effect size was attenuated after controlling for trait anxiety, indicating that general anxiety traits partially accounted for between-group differences; however, exposure type still had an independent effect.

#### 4.3.2 Between-group differences in social anxiety

H2 predicted that the solo-exposed group would have significantly higher social anxiety levels than the section group. Between-group comparison results are presented in Table 3. The solo-exposed group had higher LSAS total scores (*M* = 52.78, *SD* = 22.43) than the section group (*M* = 44.56, *SD* = 20.52), *t*(150) = 2.44, *p =* 0.016, Cohen’s *d* = 0.38, 95% CI [0.06, 0.70]. This effect size represents a small to medium effect.

**Table 3:** Between-group comparisons of MPA and social anxiety.

Variable	Solo-exposed (*n* = 74) *M* (SD)	Section (*n* = 78) *M* (SD)	*t*	*p*	Cohen’s *d* [95% CI]
K-MPAI total	98.47 (38.26)	82.20 (35.18)	2.82	0.005	0.44 [0.12, 0.77]
LSAS total	52.78 (22.43)	44.56 (20.52)	2.44	0.016	0.38 [0.06, 0.70]
LSAS-fear	28.15 (11.67)	23.82 (10.43)	2.49	0.014	0.39 [0.07, 0.71]
LSAS-avoidance	24.63 (11.58)	20.74 (10.86)	2.21	0.028	0.35 [0.03, 0.67]
STAI-T	43.89 (10.24)	41.12 (9.18)	1.82	0.071	0.28 [−0.04, 0.61]

M = mean; SD = standard deviation; CI = confidence interval. Effect size interpretation: small (*d* = 0.2), medium (*d* = 0.5), large (*d* = 0.8).

On the two LSAS subscales, the solo-exposed group scored significantly higher on the fear subscale (*M* = 28.15, *SD* = 11.67) than the section group (*M* = 23.82, *SD* = 10.43), *t*(150) = 2.49, *p* = 0.014, *d* = 0.39; the avoidance subscale also showed a significant between-group difference (solo-exposed: *M* = 24.63, *SD* = 11.58; section: *M* = 20.74, *SD* = 10.86), *t*(150) = 2.21, *p* = 0.028, *d* = 0.35. Effect size patterns were consistent across both subscales, reflecting small to medium effects.

ANCOVA results indicated that the between-group difference in LSAS total scores remained significant after controlling for covariates, *F*(1, 144) = 4.18, *p* = 0.043, η^2^*p =* 0.028.

**Figure 3:**
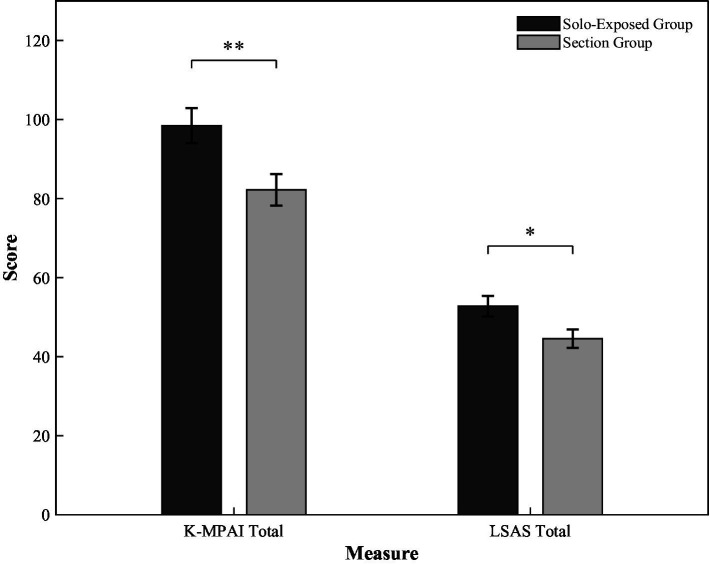
Comparison of music performance anxiety and social anxiety scores between solo-exposed and section groups. Error bars represent standard error. *p < 0.05, **p < 0.01.

Between-group comparisons of K-MPAI and LSAS scores are illustrated in Figure 3.

#### 4.3.3 Mediating effect of social anxiety

H3 predicted that social anxiety would mediate the relationship between exposure type and MPA. PROCESS macro Model 4 was used to test the mediation effect; results are presented in Table 4 and Figure 1.

**Table 4:** Mediation analysis of social anxiety.

(A) Path coefficients					
Path	*B*	SE	*t*	*p*	95% CI
a (Exposure type → LSAS)	8.22	3.47	2.37	0.019	[1.37, 15.07]
b (LSAS → K-MPAI)	0.89	0.11	8.14	<0.001	[0.68, 1.10]
c (Total effect)	16.27	5.98	2.72	0.007	[4.45, 28.09]
c’ (Direct effect)	8.95	4.52	1.98	0.049	[0.02, 17.88]

(B) Effect Decomposition (Bootstrap, 5,000 resamples).				
**Effect type**	***B***	**Boot SE**	**Boot 95% CI**	**% Effect**
Total effect (c)	16.27	5.87	[4.68, 27.86]	100%
Direct effect (c’)	8.95	4.63	[−0.18, 18.08]	55.02%
Indirect effect (a × b)	7.32	2.95	[1.86, 13.54]	44.98%

Exposure type coding: 0 = section group, 1 = solo-exposed group. Indirect effect CI not including 0 indicates significant mediation. The direct effect reached significance using standard estimation (*p* = 0.049) but the bootstrap 95% CI included zero [−0.18, 18.08]. Given that bootstrap methods are more robust to violations of normality assumptions, the direct pathway is not robustly supported. The indirect effect through social anxiety, by contrast, was significant under the same bootstrap procedure (95% CI [1.86, 13.54]), establishing social anxiety as a key mediating mechanism.

Path analysis results: (1) The effect of exposure type on social anxiety was significant (path a: *B* = 8.22, *SE* = 3.47, *t* = 2.37, *p* = 0.019), indicating higher social anxiety levels in solo-exposed players; (2) controlling for exposure type, the effect of social anxiety on MPA was significant (path b: *B* = 0.89, *SE* = 0.11, *t* = 8.14, *p* < 0.001); (3) the total effect of exposure type on MPA was significant (path c: *B* = 16.27, *SE* = 5.98, *t* = 2.72, *p* = 0.007); (4) after including the mediator, the direct effect of exposure type on MPA was attenuated but remained significant (path c’: *B* = 8.95, *SE* = 4.52, *t* = 1.98, *p* = 0.049).

Indirect effect test: Bootstrap analysis (5,000 resamples) indicated that the indirect effect through social anxiety was 7.32, with a 95% CI of [1.86, 13.54] not including zero, indicating significant mediation. The indirect effect accounted for 44.98% of the total effect.

The direct effect reached significance using standard estimation (*p* = 0.049), but the bootstrap 95% CI included zero [−0.18, 18.08], indicating that the direct pathway was not robustly supported across estimation procedures.

#### 4.3.4 Moderating effect of performance experience

H4 predicted that performance experience would moderate the relationship between exposure type and MPA, such that greater experience would attenuate between-group differences. Hierarchical regression analysis was conducted to test this hypothesis; results are presented in Table 5.

**Table 5:** Hierarchical regression analysis of performance experience moderation.

**Predictor**	**Model 1 B (SE)**	**Model 2 B (SE)**	**Model 3 B (SE)**
Step 1: Control variables			
Age	−0.42 (0.39)	−0.25 (0.41)	−0.21 (0.40)
Gender	8.56 (6.12)	7.89 (5.94)	7.45 (5.87)
Instrument type	−3.24 (6.08)	−2.87 (5.89)	−2.65 (5.82)
Step 2: Main effects			
Exposure type (A)		14.83 (5.52)**	14.56 (5.45)**
Performance experience (B)		−1.12 (0.62)	−0.98 (0.63)
Step 3: Interaction			
A × B			−1.78 (0.89)*
*R* ^2^	0.058	0.125	0.149
Δ*R*^2^	—	0.067**	0.024*
*F*	3.02*	4.15**	4.24***

*N* = 152. Exposure type: 0 = section, 1 = solo-exposed. Performance experience indexed by years of orchestra playing, mean-centered. *p < 0.05, **p < 0.01, ***p < 0.001.

Hierarchical regression results: Step 1 entered control variables (age, gender, instrument type), which explained 5.8% of the variance (*R*^2^ = 0.058, *F* = 3.02, *p* = 0.032). Step 2 entered exposure type and performance experience (both mean-centered), with a significant increase in explained variance (Δ*R*^2^ = 0.067, Δ*F* = 5.56, *p* = 0.005); the main effect of exposure type was significant (*B* = 14.83, *p* = 0.008), while the main effect of performance experience did not reach statistical significance (*B* = −1.12, *p* = 0.073). Step 3 entered the exposure type × performance experience interaction term, which further increased explained variance (Δ*R*^2^ = 0.024, Δ*F* = 4.02, *p* = 0.047), with a significant interaction effect (*B* = −1.78, *SE* = 0.89, *t* = −2.01, *p* = 0.047).

**Figure 4:**
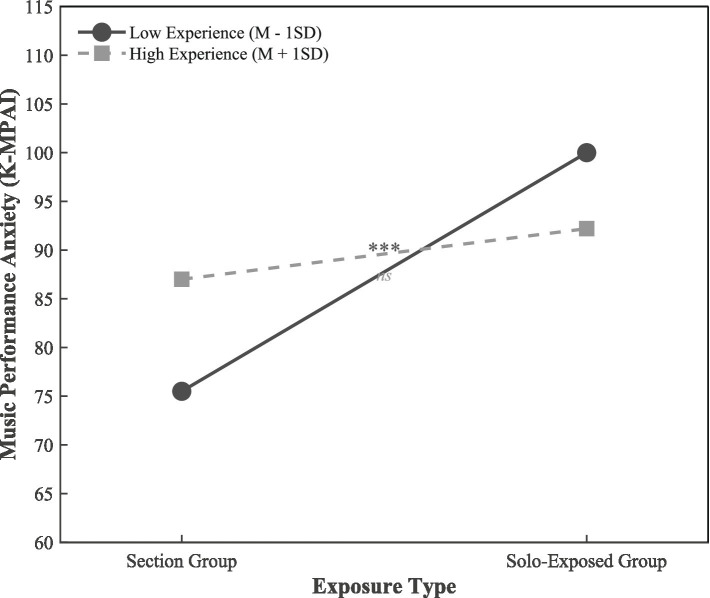
Simple slopes of the effect of exposure type on music performance anxiety at low and high levels of performance experience.

Simple slopes analysis was conducted to interpret the interaction pattern. Although the interaction effect size was small (Δ*R*^2^ = 0.024), the directional pattern was clear: among low-experience players (*M* − 1*SD*, approximately 3.2 years), solo-exposed players had significantly higher MPA than section players (*B* = 24.49, *SE* = 7.15, *t* = 3.42, *p* < 0.001); among high-experience players (*M* + 1*SD*, approximately 14.1 years), the between-group difference was attenuated and no longer significant (*B* = 5.17, *SE* = 6.83, *t* = 0.76, *p* = 0.450). The moderation pattern is illustrated in Figure 4.

**Figure 5:**
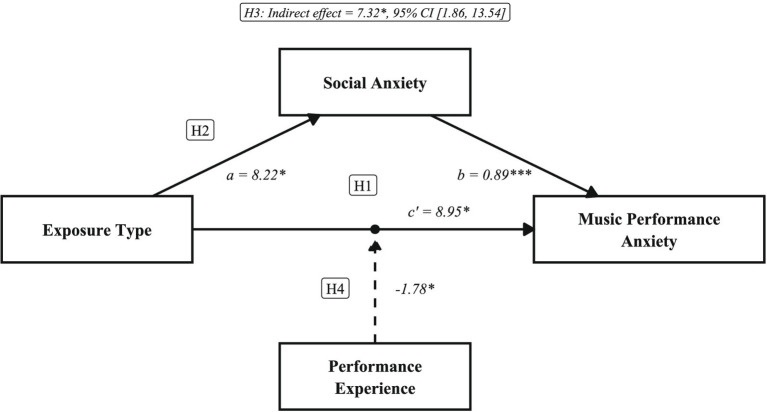
Integrated model of mediation and moderation effects with unstandardized path coefficients. *N* = 152. Unstandardized coefficients. Indirect effect = 44.98% of total effect. *p < 0.05, ***p < 0.001.

To present the complete analytical results visually, mediation and moderation effects are integrated in Figure 5.

### 4.4 Supplementary analyses

#### 4.4.1 Instrument type subgroup analysis

Between-group differences were examined separately among woodwind players (*n* = 82) and brass players (*n* = 70). Among woodwind players, the solo-exposed group had significantly higher K-MPAI scores (*M* = 100.23, *SD* = 36.85) than the section group (*M* = 81.56, *SD* = 33.42), *t*(80) = 2.43, *p* = 0.017, *d* = 0.53, 95% CI [0.09, 0.97]. Among brass players, the solo-exposed group (*M* = 96.18, *SD* = 40.12) scored higher than the section group (*M* = 83.05, *SD* = 37.68); however, this difference did not reach statistical significance, *t*(68) = 1.47, *p* = 0.146, *d* = 0.34, 95% CI [−0.14, 0.81], likely reflecting insufficient statistical power due to the smaller subgroup sample size (*n* = 70).

Two-way ANOVA results indicated a significant main effect of exposure type, *F*(1, 148) = 7.24, *p* = 0.008, η^2^*p* = 0.047; a non-significant main effect of instrument type, *F*(1, 148) = 0.35, *p* = 0.556; and a non-significant exposure type × instrument type interaction, *F*(1, 148) = 0.28, *p* = 0.598. These results suggest that although the between-group difference did not reach significance in the brass player subgroup (possibly due to smaller sample size), the overall pattern of exposure type effects on MPA was consistent across both instrument types.

#### 4.4.2 K-MPAI subscale analysis

Between-group comparisons were conducted for each of the six K-MPAI subscales (with Bonferroni correction, *α* = 0.008). Results revealed significant between-group differences on the performance-situation anxiety subscale (solo-exposed: *M* = 22.35, *SD* = 8.12; section: *M* = 17.64, *SD* = 7.58), *t*(150) = 3.83, *p* < 0.001, *d* = 0.60, 95% CI [0.27, 0.93]; and on the worry/rumination subscale (solo-exposed: *M* = 19.47, *SD* = 7.89; section: *M* = 15.82, *SD* = 7.23), *t*(150) = 3.08, *p* = 0.002, *d* = 0.48, 95% CI [0.16, 0.80].

The proximal somatic anxiety subscale showed a small effect that did not survive Bonferroni correction (*t* = 2.15, *p* = 0.033, *d* = 0.35, 95% CI [−0.03, 0.68]). The early relationship context (*t* = 0.89, *p* = 0.374, *d* = 0.16, 95% CI [−0.16, 0.48]), psychological vulnerability (*t* = 1.43, *p* = 0.155, *d* = 0.23, 95% CI [−0.09, 0.55]), and depression/hopelessness (*t* = 1.12, *p* = 0.264, *d* = 0.22, 95% CI [−0.10, 0.54]) subscales showed small, non-significant effects.

### 4.5 Sensitivity analysis: exclusion of beta-blocker users

Given that beta-blocker use was significantly more prevalent in the solo-exposed group (28.4%) than in the section group (14.1%), a sensitivity analysis was conducted to examine whether the primary findings were robust after excluding all beta-blocker users (remaining sample: *n* = 120; solo-exposed *n* = 53, section *n* = 67).

Between-group differences remained significant for both K-MPAI total scores (solo-exposed: *M* = 95.23, *SD* = 37.82; section: *M* = 80.45, *SD* = 34.56), *t*(118) = 2.28, *p* = 0.024, *d* = 0.41, and LSAS total scores (solo-exposed: *M* = 51.56, *SD* = 21.87; section: *M* = 43.78, *SD* = 20.05), *t*(118) = 2.01, *p* = 0.047, *d* = 0.37. Effect sizes were slightly attenuated compared to the full sample (K-MPAI *d*: 0.44 → 0.41; LSAS *d*: 0.38 → 0.37), consistent with the removal of a disproportionate number of higher-anxiety individuals from the solo-exposed group.

The mediation analysis was also replicated in this reduced sample. The indirect effect of exposure type on MPA through social anxiety remained significant (indirect effect = 6.89, Bootstrap 95% CI [1.42, 13.12]). These results indicate that the primary findings were not substantially driven by differential beta-blocker use between groups.

## 5 Discussion

### 5.1 Summary of findings

The present study aimed to examine the association between exposure level and MPA among wind instrumentalists in orchestra settings and to explore the mediating role of social anxiety and the moderating role of performance experience. Based on a sample of 152 wind instrumentalists, results supported all four hypotheses: (1) solo-exposed players had significantly higher MPA levels than section players (*d* = 0.44); (2) solo-exposed players had significantly higher social anxiety levels than section players (*d* = 0.38); (3) social anxiety significantly mediated the relationship between exposure type and MPA, accounting for 44.98% of the total effect; (4) performance experience moderated the relationship between exposure type and MPA, with the between-group difference being attenuated among highly experienced players.

### 5.2 Interpretation and theoretical implications

#### 5.2.1 Exposure level and music performance anxiety

The present study found that solo-exposed wind instrumentalists had significantly higher MPA levels than section players, consistent with prior research on differences between solo and ensemble contexts (Papageorgi et al., 2013; Robson and Kenny, 2017). However, the unique contribution of this study lies in revealing that even within the same orchestra environment, performers face significantly different anxiety risks depending on their position and responsibilities. Section principals and those assigned solo passages, though nominally “orchestra players,” may have psychological experiences more closely resembling those of soloists, providing a new perspective for understanding the context-specificity of MPA. This finding is consistent with the self-presentation model (Schlenker and Leary, 1982) and the cognitive model of social anxiety (Rapee and Heimberg, 1997): solo-exposed positions increase both the visibility of individual performance and the perceived probability of detectable error, thereby activating the core cognitive mechanisms that generate social evaluative anxiety.

The effect size in the present study (*d* = 0.44) represents a medium effect, smaller than effect sizes reported in some studies comparing pure solo versus ensemble contexts. This difference likely reflects the continuous nature of within-orchestra exposure level differences—even solo-exposed players remain embedded within the overall orchestra framework, which to some extent buffers the pressure associated with purely solo performance. Importantly, the practical significance of these differences should not be underestimated despite the moderate effect sizes. When evaluated against clinical benchmarks, 41.9% of solo-exposed players scored at or above the suggested K-MPAI clinical threshold (≥105; Kenny, 2011), compared to 25.6% of section players—a substantial difference in the proportion of musicians experiencing clinically significant anxiety. Similarly, the solo-exposed group mean on the LSAS (*M* = 52.78) approached the threshold for marked social anxiety, while the section group (*M* = 44.56) remained in the moderate range, indicating a qualitative difference in clinical severity despite a relatively modest quantitative gap. Furthermore, the significantly higher rate of beta-blocker use in the solo-exposed group (28.4% vs. 14.1%) provides behavioral evidence that the observed anxiety differences translate into tangible consequences for musicians’ daily professional lives. It is also worth noting that the two groups share the same organizational environment, training background, and rehearsal schedules; the only systematic difference is their degree of performance exposure. Detecting a medium effect under such highly homogeneous conditions suggests a meaningful and robust phenomenon—one that would likely be larger if more heterogeneous comparison groups were studied.

#### 5.2.2 Mediating mechanism of social anxiety

The present study established the mediating role of social anxiety in the relationship between exposure type and MPA, providing important evidence for understanding the psychological mechanisms of MPA. According to Kenny (2011) three-type taxonomy of MPA, focal MPA significantly overlaps with social anxiety disorder, sharing fear of negative evaluation as a core feature. The present findings suggest that higher MPA among solo-exposed players is associated with stronger concerns about social evaluation that may be activated by their role responsibilities.

The mediating effect accounted for 44.98% of the total effect, suggesting that social anxiety is an important but not sole mediating variable. The direct effect remained significant (although the bootstrap 95% CI for the direct effect included zero, suggesting that the direct pathway is not robustly supported), indicating that exposure type may also influence MPA through other pathways, such as higher technical demands, greater responsibility pressure, or stronger perfectionism tendencies. This is consistent with Barlow (2000) triple vulnerability model of anxiety, which posits that MPA development involves interactions among biological, psychological, and situation-specific factors.

#### 5.2.3 Protective role of performance experience

The present study found that performance experience significantly moderated the relationship between exposure type and MPA, specifically manifesting as: among low-experience players, MPA differences between solo-exposed and section players were significant with a large effect size; among high-experience players, this difference was no longer significant. This finding is broadly consistent with predictions from habituation theory and self-efficacy theory (Bandura, 1977), which suggest that repeated successful performance experiences may reduce threat perception and enhance confidence in coping with high-pressure contexts.

This protective role of experience is consistent with recent findings by Yang et al. (2025), who reported that psychological resilience moderated the link between performance competence and anxiety, suggesting that accumulated psychological resources—whether through experience or resilience—may be associated with attenuated anxiety in demanding performance contexts. It is also worth noting that the present study did not account for potential differences in musical characteristics between solo-exposed passages and section parts (e.g., technical difficulty, melodic prominence, textural exposure). Cui et al. (2025) demonstrated that participant characteristics and musical characteristics interact to influence physiological stress during opera performances, highlighting the importance of disentangling “degree of exposure” from “passage difficulty” in future research.

However, this finding must be interpreted with considerable caution. The moderating effect explained only 2.4% of additional variance in MPA beyond the main effects (Δ*R*^2^ = 0.024), and the interaction term was close to the conventional significance threshold (*p* = 0.047), indicating limited practical explanatory power. The cross-sectional design further constrains interpretation, as selection effects cannot be ruled out—high-anxiety solo-exposed players may exit those positions earlier, leaving only individuals with higher anxiety tolerance in the high-experience group. Additionally, the accumulation of experience may be accompanied by various unmeasured psychological changes such as cognitive reappraisal and coping strategy development, which the present study did not disentangle. Therefore, while the moderation pattern is theoretically plausible and directionally informative, it should be regarded as preliminary evidence requiring replication with larger samples and longitudinal designs.

#### 5.2.4 K-MPAI subscale difference patterns

Supplementary analyses revealed that between-group differences were concentrated on the performance-situation anxiety and worry/rumination subscales, with no significant differences on early relationship context, psychological vulnerability, or depression/hopelessness subscales. This pattern has important theoretical implications: it suggests that anxiety differences between solo-exposed and section players are primarily situation-driven rather than stemming from early developmental experiences or general psychological vulnerability. In other words, current performance situation characteristics (rather than individuals’ long-standing traits) shape between-group anxiety differences, providing a theoretical basis for situation-focused interventions.

### 5.3 Practical implications

The findings of the present study have several implications for music education and performer mental health practice.

First, orchestra managers and conductors should recognize that section principals and performers assigned solo tasks face elevated psychological pressure. For newly appointed principals or those undertaking major solos for the first time, additional psychological support should be considered, such as gradual exposure training, peer support systems, or access to professional counseling resources.

Second, given the mediating role of social anxiety, intervention strategies targeting social anxiety may be particularly valuable for alleviating MPA in solo-exposed players. Cognitive restructuring techniques targeting fear of negative evaluation from cognitive behavioral therapy (CBT) and defusion techniques from acceptance and commitment therapy (ACT) may be especially applicable to this population. A recent systematic review has synthesized evidence for various therapeutic interventions for MPA, providing guidance for selecting appropriate treatment modalities (Kinney et al., 2025).

Third, although the moderating effect of performance experience was small in magnitude, the observed pattern tentatively suggests that progressively challenging performance opportunities for solo-exposed players early in their careers may contribute to long-term psychological adaptation. This implication should be considered preliminary and warrants further investigation through longitudinal and intervention studies before informing policy recommendations. Music conservatories could consider systematically incorporating gradual performance training into curricula to help students accumulate sufficient high-exposure experience before becoming professional performers. Innovative approaches such as virtual reality exposure therapy have shown promise as adjuncts to traditional methods (Bellinger et al., 2023). Given that wind performance is highly dependent on breath control and that anxiety directly disrupts respiratory function, breathing-based interventions may be particularly well-suited for this population. A randomized controlled trial demonstrated that a single session of biofeedback training significantly reduced performance anxiety and improved heart rate variability in musicians (Wells et al., 2012), pointing to the potential value of incorporating somatic regulation techniques into intervention programs for wind instrumentalists.

Finally, the significantly higher beta-blocker use rate in the solo-exposed group (28.4%) compared to the section group (14.1%) reflects this population’s greater need for pharmacological assistance. This suggests that relevant practitioners should attend to medication dependency risks and explore the accessibility of non-pharmacological interventions. Importantly, our sensitivity analysis demonstrated that the observed group differences in MPA and social anxiety, as well as the mediating role of social anxiety, remained significant after excluding all beta-blocker users, indicating that the primary findings were robust to the potential confounding influence of pharmacological management.

### 5.4 Limitations

The present study has several limitations. First, the cross-sectional design limits causal inference. Although this study hypothesized that exposure type influences anxiety levels, reverse causality is equally possible—low-anxiety individuals may be more likely to be selected as principals or actively seek solo opportunities. Future research could employ longitudinal designs to track anxiety changes before and after position transitions.

Second, the sample comprised predominantly mainland Chinese performers; cultural factors may influence the cross-cultural generalizability of findings. Concerns about “losing face” in East Asian cultural contexts may amplify the role of social anxiety, whereas MPA mechanisms in Western individualistic cultures may differ. Studies with Chinese music teachers have begun to explore these cultural nuances (Cui et al., 2024).

Third, although the operationalization of exposure level was carefully considered, some degree of subjectivity remains. Some performers may possess characteristics of both groups (e.g., non-principals who occasionally perform solos), and ambiguity in classification boundaries may lead to effect size underestimation.

Fourth, this study relied on self-report measures, which may be subject to social desirability and recall biases. Future research could incorporate physiological indicators (e.g., cortisol levels, heart rate variability) and behavioral observations (e.g., actual performance quality assessments) for multimodal validation.

Fifth, although the sample size met statistical power requirements, statistical power was reduced for subgroup analyses (e.g., brass players), potentially resulting in some true effects not being detected.

### 5.5 Future directions

Based on the findings and limitations of the present study, future research may explore the following directions.

First, longitudinal tracking designs could examine dynamic changes in MPA as performers transition from section to solo-exposed positions (e.g., promotion to principal), establishing more reliable causal inferences.

Second, other potential mediating variables could be explored, such as perfectionism, self-efficacy, and coping strategies, to construct a more complete model of MPA mechanisms. Moderated mediation models could also be tested to examine whether performance experience moderates the mediating effect of social anxiety.

Third, cross-cultural comparative studies could test the robustness of the present findings across different cultural contexts and explore culture-specific protective or risk factors.

Fourth, intervention studies could be designed to test the effectiveness of targeted interventions for solo-exposed players (e.g., social anxiety-focused CBT, gradual exposure training).

Fifth, the research scope could be extended to other instrument groups (e.g., strings, percussion) and other performance contexts (e.g., chamber music, concerto solos) to test the generalizability of the present findings.

Sixth, although the Chinese K-MPAI used in the present study was developed through rigorous translation-back-translation procedures with the original author’s permission and demonstrated high internal consistency in our sample (Cronbach’s *α* = 0.92), a comprehensive psychometric validation (e.g., confirmatory factor analysis of the six-factor structure, test–retest reliability, measurement invariance across subgroups) was beyond the scope of the present study. Future research should establish the full psychometric properties of the Chinese K-MPAI in larger and more diverse Chinese-speaking musician samples, including formal comparison with the four-factor 26-item Chinese solution recently reported by Pan et al. (2025) in vocal-music students.

Finally, future research may benefit from adopting data-informed approaches to identify distinct profiles of performance anxiety among musicians. For example, clustering or latent profile analysis based on participants’ response patterns could reveal qualitatively different anxiety subtypes that may require differentiated intervention strategies. Such approaches have been productively applied in related artistic domains—for instance, computational classification methods have been used to detect distinct levels of theatrical performance anxiety among students (Mastrothanasis et al., 2023)—and could be extended to orchestral musicians to better capture the heterogeneity of MPA identified in the present study.

## 6 Conclusion

Using a sample of 152 wind instrumentalists, the present study systematically examined the association between exposure level and MPA in orchestra settings and the psychological mechanisms underlying this association. Results indicated that solo-exposed players (section principals or those performing solo passages) had significantly higher MPA and social anxiety levels than section players, and these differences persisted after controlling for demographic variables and trait anxiety. Social anxiety significantly mediated the relationship between exposure type and MPA, accounting for 44.98% of the total effect, suggesting that high-exposure positions are associated with elevated MPA, potentially through stronger concerns about social evaluation. Performance experience showed a protective moderating pattern in this relationship: among less experienced players, the between-group difference was significant; among highly experienced players, this difference was no longer significant. Additionally, between-group differences were primarily evident in performance-situation anxiety and worry/rumination—situation-related dimensions—rather than early experiences or psychological vulnerability—trait-related factors. The findings of the present study extend theory on the context-specificity of MPA and provide empirical reference for orchestra managers to identify high-risk performers and for mental health practitioners to develop targeted interventions.

## Data Availability

The original contributions presented in the study are included in the article/supplementary material, further inquiries can be directed to the corresponding author.
